# Dual dimensions of artificial intelligence use among medical academia: related knowledge, attitudes and ethical concerns, a national survey, 2025

**DOI:** 10.1038/s41598-026-54681-z

**Published:** 2026-06-04

**Authors:** Doaa Ibrahim Omar, Salman Althobaiti, Basma M. Hani, Shaima Adel Elsayed Ali, Mai Magdy Anwer

**Affiliations:** 1 Department of Community, Environmental and Occupational Medicine, Faculty of Medicine, Benha University, Benha, Egypt; 2 Department of Family, Community and Medical Education, Faculty of Medicine, Taibah University, Madinah, Saudi Arabia

**Keywords:** Artificial intelligence, Attitude, Knowledge, Medical education, Medical practice, Concerns, Health care, Psychology, Psychology

## Abstract

AI integration into medical education and practice has its benefits and risks. This national web-based cross-sectional survey on Egyptian medical staff and students aimed to assess knowledge, attitudes, and concerns regarding the use of AI. This study comprised 2765 medical students and 500 medical staff, with a mean age of 20.8 and 29.9 years, respectively, and higher percentages of females among both groups. Medical students demonstrated satisfactory knowledge of AI compared to medical staff (*p* < 0.001). Unfortunately, the majority of both groups (80.4% of staff and 81.6% of students) expressed negative attitudes toward AI use. Male participants had significantly higher attitude scores than females in both groups. Knowledge score and gender were significant predictors of attitude towards AI among medical staff (*p*<0.005), while gender was a significant predictor of attitude scores among medical students (*p* < 0.001). The total AI usage score in this study was higher among students than staff, particularly for idea generation. Medical staff demonstrated a slightly higher total concern score regarding the use of AI in medical education and practice compared with students. The results emphasize the necessity of engagement, focused education and training, compatible solutions, uniform standards, guidelines, and the smooth incorporation of AI into medical education and clinical practice.

## Background

Artificial Intelligence (AI) is a system that analyses the environment and makes decisions based on human intelligence to accomplish specific objectives^1^. Various sectors, including economics, manufacturing, education, and healthcare, have adopted AI. In the medical field, AI is rapidly evolving with the potential to revolutionize various aspects of healthcare, including diagnostics, treatment planning, and personalized medicine^2^. Physicians, academics, and clinicians are all interested in AI, a game-changing development in healthcare^3^. The use of AI in medicine is expected to significantly impact patient care, medical research, and health systems. In medical education, AI technologies such as machine learning (ML), natural language processing (NLP), and generative AI tools (e.g., GPT-based systems) are transforming both medical education content and processes. As technology continues to advance, it is crucial to examine the effect of these developments on all aspects of medical education, from admissions to curricula, teaching, and assessment^4^. Beyond education, AI has demonstrated significant potential in public health applications, including the detection of disease outbreaks using data from search engine queries and social media. Notably, Google forecasted influenza epidemics, whereas the Chinese Academy of Sciences used multi-source information fusion to simulate the COVID-19 pandemic^5,6^. Despite these advances, several misconceptions persist regarding AI in healthcare, including the belief that AI will replace physicians or that advanced programming skills are required for its use. In reality, AI is intended to augment rather than replace clinical decision-making, and its effective use requires conceptual understanding rather than technical expertise^5,6^. Artificial Intelligence is becoming increasingly prevalent, with many applications in medical education and medical practice. However, many barriers can hinder AI implementation, and ethical issues should be taken into consideration too^7^. To fully achieve the potential of AI in healthcare, four major ethical issues must be carefully addressed during the incorporation of AI in healthcare: informed consent to use data, safety and transparency, algorithmic fairness and biases, and finally, data privacy. Also, one of the major concerns about the use of AI technologies is data privacy and security, and technological addiction among users^8^. Moreover, at a time when developed nations invest significantly in AI research, developing nations face challenges in AI implementation in education and research due to its high cost^9^.

In the available Egyptian literature, to the best of the authors’ knowledge, all the Egyptian studies have explored AI-related knowledge, perceptions, and or attitudes among medical students at single institutions using relatively small sample sizes^7,10–12^. So the previous studies were generally confined to a single institution and a single field, either medical education or clinical medical practice. Furthermore, most studies have targeted medical students, with relatively limited inclusion of medical staff or comparative analyses between different professional groups. There is a lack of comprehensive studies that simultaneously assess multiple dimensions of AI use. To address these gaps, the current study aimed to assess AI-related knowledge, patterns of use, attitudes, and concerns among both medical students and medical staff. By adopting a multidimensional approach and including diverse participant groups, this study provides a more comprehensive understanding of AI integration in both educational and clinical contexts. The study also adopted a nationally representative approach with a large-sized sample supporting the validity, generalizability of the reported data, and constitutes a key aspect of its originality and contribution to the literature.

## Methodology

### Study design, duration, and participants

This national cross-sectional study involved Egyptian medical staff members and students, during the period from 1st of April 2025, to the 1st of Sep 2025.

All methods were performed in accordance with the relevant guidelines and regulations.

### Sample size

The sample size was calculated using the following formula, $\:\text{}n\mathrm{=}\frac{{Z}^{2}P\left(1\mathrm{-}P\right)}{{d}^{2,}}$where n is the calculated sample size, z equals 1.96 for a 95% CI, d equals 0.05, and p is the proportion of positive attitudes toward AI tools, assumed to be 50%. The calculated sample size was 385 participants from each group, so the total calculated sample size for both groups was 770.

### Sampling strategy

Stratification according to the type of the medical faculty, either governmental, national, or private, was done. In Egypt, there are 27 governmental, 32 national, and 20 private medical Universities during the academic year 2024–2025^13^. Students from all targeted faculties were asked to participate in the study through the data collectors, a mixed sampling technique was employed. Both convenient and snowball sampling techniques were used to reach as many participants as possible, through all communication channels.

### The study tools and data collection process

After an extensive literature review, the questionnaire used in this study was adapted from previously published, open-access studies that had validated the tool^7,11,14,15^.

The questionnaire was composed of 7 sections as follows.

The 1st section: sociodemographic characteristics: age, gender, level, Grade Point Average (GPA), residence, governorate, and university type. The 2nd section: Knowledge of AI technologies, adapted from^13^, and composed of 7 questions, responses are yes, no, and not sure. The correct answer scored as 1, and the incorrect or don’t know answer scored as zero; the maximum score was 7, and the minimum score was zero. The level of knowledge is considered satisfactory if ≥ 50% of the total score is achieved. 3rd section: Attitude towards AI tools usage, adapted from^13^, and consisted of 10 questions, on a five-point Likert scale, where strongly agree scores as 5 and strongly disagree scores as 1, with reverse scoring of the negative sentences, the maximum score is 50, and the minimum score is 10; the higher the score, the more positive the attitude toward AI usage. Attitude is considered positive when ≥ 80% of the total score is achieved^11^.

4th section: Perspectives on AI tools’ output, adapted from^14^, and composed of 6 questions, on a five-point Likert scale, where strongly agree scores as 1 and strongly disagree scores as 5, the maximum score was 30, and the minimum score was 6. The higher the score means more positive perspectives towards AI output.

5th section: Patterns of Use of AI tools in medical education and research, adapted from^14^, on a five-point Likert scale ranging from never (scores as 1) to very often (scores as 5), the maximum score was 35, and the minimum score was 7 (the higher the score, the more frequent the pattern of usage of AI in medical education and research). 6th section: Concerns towards AI technologies in medical education, adapted from^15^.7th section: Concerns towards AI technologies in medical practice, adapted from^7^, where agree scored as 3 and disagree scored as 1 (the higher the score, the more concerns).

### Validation of the study tool

The study’s tools were obtained from previously published studies that had undergone validation. Integration of previously validated questionnaires from separate studies into a single tool, to cover all dimensions aimed to be explored in our study^7,11,14,15^, these tools were validated in the original studies by a panel of 3–4 experts, pilot tested on 30–40 participants, and internal consistency was measured using Cronbach’s alpha. However, in the current study, a jury of 3 experts (2 public health experts and 1 occupational health expert) confirmed face and content validity of the questionnaire. A pilot study on 30 medical students and 10 medical staff was conducted to assess face validity (comprehension and clarity of the questionnaire). Minor linguistic adaptations were made to the tool to ensure contextual appropriateness and clarity.

Reliability (internal consistency) of the questionnaire was tested by calculating Cronbach’s alpha; it was 0.70 for knowledge, 0.70 for attitude, 0.74 for perspective on AI output, 0.80 for the pattern of use of AI tools, 0.75 for concerns of AI use in medical education, and 0.73 for concerns of AI use in medical practice. Thus, the overall Cronbach’s alpha was 0.74.

### Methods of data collection

The Google form of the questionnaire was shared with medical staff and students through email, WhatsApp, Facebook, and Telegram. Medical students and staff from Benha Faculty of Medicine, and data collectors were instructed to distribute the Google form of the questionnaire to their network of friends and medical colleagues in governmental, national, and private medical faculties in Egypt through all social media platforms (WhatsApp, Telegram, Instagram, Facebook, and Twitter). Three reminders were sent to non-respondents, with one weak interval, to involve as many participants as possible.

## Results

This study included 500 medical staff and 2,765 medical students, with a higher proportion of females in both groups. The mean age was 29.93 (SD = 8.67) years for the medical staff and 20.82 (SD = 1.98) years for the students. The majority of both groups were affiliated with public universities. The highest proportion of students was in level 2 (33.9%) (Table 1).

**Table 1:** Sociodemographic characteristics among the study population (*N* = 3265).

Sociodemographic characteristics		Medical staff (*N* = 500) *n* (%)	Medical students (*N* = 2765) *n* (%)
Gender	Male	189 (37.8%)	1124 (40.7%)
	Female	311 (62.2%)	1641 (59.3%)
Age (years)	Mean (SD)	29.93 (8.67)	20.82 (1.98)
Residence	Urban	318 (63.6%)	1838 (66.5%)
	Rural	182 (36.4%)	927 (33.5%)
Nationality	Egyptian	408 (81.6%)	1610 (58.2%)
	Not Egyptian	92 (18.4%)	1155 (41.8%)
Type of university	Governmental	434 (86.8%)	2532 (91.6%)
	National	41 (8.2%)	151 (5.5%)
	Private	25 (5.0%)	82 (3.0%)
Governmental Universities	Benha Zagazig Kafr-Elsheikh Minufya Tanta Ain Shams Cairo Al-Azhar Helwan Alexandria Mansoura Fayoum Suez Canal New Valley Port Said Assiut Minia	150 (34.6%) 67 (15.4%) 21 (4.8%) 19 (4.4%) 18 (4.1%) 13 (3.0%) 12 (2.8%) 16 (3.7%) 12 (2.8%) 15 (3.5%) 14 (3.2%) 15 (3.5%) 10 (2.3%) 13 (3.0%) 14 (3.2%) 15 (3.4%) 10 (2.3%)	1000 (39.5%) 222 (8.8%) 71 (2.8%) 78 (3.1%) 97 (3.8%) 122 (4.8%) 122 (4.8% 89 (3.5%) 98 (3.9%) 111 (4.4%) 86 (3.4%) 59 (2.3%) 79 (3.1%) 63 (2.5%) 61 (2.4%) 91 (3.6%) 83 (3.3%)
Specialty	Academic	204 (40.8%)	–
	Medicine	214 (42.6%)	
	Surgery	82 (16.6%)	
Level of medical education	Level 1	–	646 (23.3%)
	Level 2		937 (33.9%)
	Level 3		528 (19.1%)
	Level 4		312 (11.3%)
	Level 5		231 (8.4%)
	Internship		111 (4.0%)

The results of this study identified that, among the medical staff, males had significantly higher perceived mean knowledge scores than females (5.22 vs. 4.83, *p* = 0.012), whereas no gender difference was observed among students.

Mean attitude scores were significantly (*p* < 0.001) higher in males compared to females for both staff and students. The type of university was significantly associated with staff attitude scores (*p* < 0.001), as the highest mean attitude score was among private universities. The perceived knowledge level showed a highly statistically significant association with attitude score among both medical staff and students. Staff and students with a good perceived knowledge level showed more positive attitude scores (*p* < 0.001) than those with a poor perceived knowledge level. Age, residence, and nationality were not significantly associated with knowledge or attitude scores in either group. Overall, 57.6% of staff and 69.5% of students achieved good knowledge scores (> 50%), while only 19.6% of staff and 18.4% of students demonstrated positive attitudes (≥ 80%) toward AI use. A higher proportion of medical students had good perceived knowledge (69.5%) when compared to medical staff (57.6%) (Table 2).

**Table 2:** Perceived knowledge and attitude towards Artificial Intelligence in relation to sociodemographic characteristics of the studied population (*N* = 3265).

The variables		Knowledge score		Attitude score	
		Medical staff Mean (SD) 4.98 (1.68)	Medical students Mean (SD) 5.12 (1.74)	Medical staff Mean (SD) 35.29 (5.29)	Medical students Mean (SD) 35.13 (5.04)
Gender	Male	5.22 (1.62)	5.12 (1.72)	36.55 (6.01)	35.76 (5.23)
	Female	4.83 (1.70)	5.13 (1.76)	34.52 (4.65)	34.70 (4.82)
p-value		0.012*	0.894	< 0.001**	< 0.001**
Age (years)	r	–0.069	0.023	0.031	0.030
p-value		0.122	0.250	0.495	0.117
Residence	Urban	5.04 (1.64)	5.11 (1.73)	35.6 (5.26)	35.15 (5.10)
	Rural	4.86 (1.74)	5.15 (1.78)	34.74 (5.32)	35.02 (4.83)
p-value		0.250	0.610	0.082	0.251
Nationality	Egyptian	4.98 (1.71)	5.18 (1.73)	35.44 (5.22)	35.25 (5.08)
	Non-Egyptian	4.96 (1.54)	5.05 (1.76)	34.60 (5.59)	34.96 (4.90)
p-value		0.924	0.072	0.168	0.134
Type of university	Governmental	4.96 (1.68)	5.12 (1.74)	35.18 (5.19)	35.06 (4.99)
	National	4.90 (1.61)	5.19 (1.75)	33.61 (5.13)	35.63 (5.43)
	Private	5.48 (1.75)	4.98 (1.75)	39.84 (5.15)	35.61 (4.73)
p-value		0.303	0.718	< 0.001**	0.431
Perceived knowledge level	Poor	–	–	34.40 (4.81)	33.58 (5.01)
	Good	–	–	35.94 (5.54)	35.81 (4.89)
p-value		–	–	**0.001**	**< 0.001**

		Medical staff (N.=500) N (%)		Medical students (N.=2765) N (%)	
Perceived knowledge level	Poor	212 (42.4%)		842 (30.5%)	
	Good	288 (57.6%)		1923 (69.5%)	
*χ*^2^ (p-value)		27.651 (< 0.001)**			
Attitude level	Positive	98 (19.6%)		509 (18.4%)	
	Negative	402 (80.4%)		2256 (81.6%)	
*χ*^2^ (p-value)		0.397 (0.529)			

r= Pearson’s Correlation Coefficient, *χ*^2^ = Chi Squared test.

*Significant at ≤ 0.05. **Highly significant at ≤ 0.001.

A multivariate linear regression analysis was conducted to identify predictors of knowledge score among medical staff and students. The overall model for medical staff was statistically significant (F = 2.212, *p* = 0.025) but explained only 1.9% of the variance in knowledge score (adjusted R² = 0.019). Age ang gender emerged as independent predictors, with younger medical staff having significantly higher knowledge score (B = − 0.020, 95% CI: −0.040 to − 0.001, *p* = 0.037) and female medical staff having significantly lower knowledge scores compared to males (B = − 0.372, 95% CI: −0.692 to − 0.052, *p* = 0.023). On the other hand, the overall model of knowledge score among medical students was not statistically significant (*p* = 0.636). Nevertheless, given the very limited explanatory power of the model, this association should be interpreted with caution, as it is unlikely to be of practical significance.

A multiple linear regression analysis was performed to examine factors associated with the attitude score among medical staff. The model reached statistical significance (F = 8.960, *p* < 0.001) and demonstrated modest explanatory power (adjusted R^2^= 0.126). Gender, private universities, and concern scores regarding medical education and research and medical practice emerged as significant independent predictors, as female medical staff have lower attitude scores than male (B= -2.078, 95%CI: –3.036 to –1.120, *p* < 0.001), medical staff of private universities have higher attitude score (B = 4.738, 95%CI: 2.105 to 7.371, *p* < 0.001), and higher concern scores regarding medical education was a significant predictor for high attitude scores (B = 0.388, 95%CI: 0.227 to 0.549, *p* < 0.001). Meanwhile, a lower score of concerns regarding medical practice was a significant predictor of a higher attitude score among medical staff (B = -0.181, 95%CI: -0.354 to -0.008, *p* = 0.040). Among medical students a multiple linear regression analysis was performed to identify predictors of Attitude score. The model was statistically significant (F = 14.666, *p* < 0.001) and explained only 4.8% of the variance (adjusted R^2^= 0.048). Gender and concern scores regarding medical education and research were significant predictors, as female medical students have lower attitude scores than male (B = − 1.170, 95% CI: −1.570 to − 0.770, *p* < 0.001) and higher concern scores regarding medical education and research was a significant predictor for high attitude scores (B = 0.291, 95%CI: 0.216 to 0.366, *p* < 0.001) However, these findings should also be interpreted with caution given the limited explanatory power of the model (Table 3).

**Table 3:** Multiple linear regression analysis of knowledge and attitude scores towards Artificial Intelligence among the study population (*N* = 3265).

	Medical staff (*N* = 500)			Medical students (*N* = 2765)		
	B	*p* value	95% CI	B	*p* value	95% CI
*Knowledge score*						
Age (years)	–0.020	0.037*	–0.040 to –0.001	0.022	0.225	–0.013–0.057
Gender (ref: male)	–0.372	0.023*	–0.692 to –0.052	0.005	0.943	–0.137–0.148
Residence (ref: urban)	–0.184	0.249	–0.497–0.129	0.027	0.720	–0.120–0.173
Nationality (ref: Egyptian)	–0.212	0.377	–0.682–0.259	–0.128	0.077	–0.269–0.014
Type of university (ref: National)	–	–	–	–	–	–
Governmental	0.051	0.873	–0.579–0.681	–0.077	0.617	–0.380–0.226
Private	0.601	0.182	–0.283–1.485	–0.229	0.376	–0.736–0.278
Concerns regarding the medical education score	0.049	0.076	–0.005–0.103	0.010	0.456	–0.017–0.037
Concerns regarding medical practice score	–0.020	0.500	–0.078–0.038	–0.007	0.643	–0.037–0.023
*Attitude score*						
Age (years)	–0.020	0.501	–0.077–0.038	0.071	0.162	–0.028–0.170
Gender (ref: male)	–2.078	< 0.001**	–3.036 to –1.120	–1.170	< 0.001**	–1.570 to –0.770
Residence (ref: urban)	–0.695	0.144	–1.627–0.238	–0.061	0.771	–0.471–0.349
Nationality (ref: Egyptian)	–0.842	0.238	–2.242–0.558	–0.260	0.200	–0.657–0.137
Type of university (ref: National)	–	–	–	–	–	–
Governmental	0.413	0.665	–1.461–2.286	–0.634	0.143	–1.482–0.215
Private	4.738	< 0.001**	2.105–7.371	–0.138	0.849	–1.559–1.282
Knowledge score	0.182	0.176	–0.082–0.445	0.067	0.239	–0.044–0.178
Concerns regarding medical education score	0.388	< 0.001**	0.227–0.549	0.291	< 0.001**	0.216–0.366
Concerns regarding medical practice score	–0.181	0.040*	–0.354 to –0.008	–0.002	0.954	–0.086–0.081

All predictors were selected based on their theoretical relevance to the study and to look for potential confounding factors.

B = Unstandardized B coefficient, CI = Confidence Interval for unstandardized regression coefficient.

*Significant at ≤ 0.05. **Highly significant at ≤ 0.001.

### Perspectives on AI tools’ output among the studied population

**Fig. 1:**
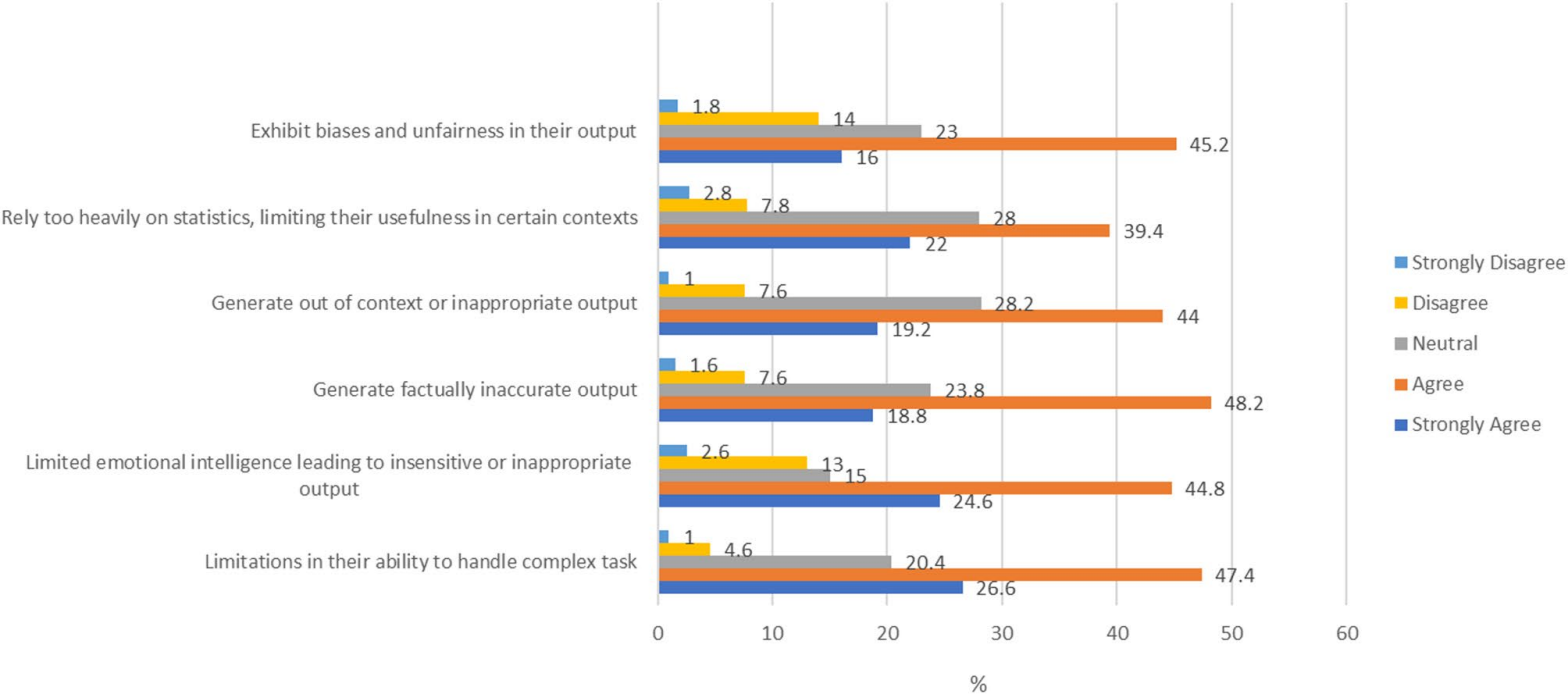
Bar chart showing the perspectives on Artificial Intelligence tools’ output among the medical staff (*N* = 500).

All mean scores are between 2.06 and 2.55, which is closer to “Agree” than “Disagree”. The highest mean was for: “AI tools can exhibit biases and unfairness in their output”. While the lowest mean was for: “AI tools have limitations in their ability to handle complex tasks”. Across all items, medical staff showed slightly negative perspectives, as reflected in their lower mean scores. Statistically significant differences between medical staff and students were observed in four items: limitations in handling complex tasks (*p* < 0.001), factually inaccurate output (*p* = 0.002), out-of-context or inappropriate output (*p* = 0.001), and bias/unfairness (*p* = 0.002) (Table 4) (Fig. 1).

**Table 4:** Perspectives on Artificial Intelligence tools’ output among the study population (*N* = 3265).

Perspectives on AI tools’ output	Medical staff (Mean (SD)	Medical students (Mean (SD)	Test of significance	*p* value
AI tools can exhibit biases and unfairness in their output	2.40 (0.98)	2.55 (0.94)	t = 3.125	0.002**
AI tools may rely too heavily on statistics, which can limit their usefulness in certain contexts	2.30 (0.99)	2.37 (0.89)	t = 1.457	0.146
AI tools can generate output that is out of context or inappropriate	2.27 (0.89)	2.41 (0.89)	t = 3.271	0.001**
AI tools can generate factually inaccurate output	2.25 (0.90)	2.38 (0.88)	t = 3.086	0.002**
AI tools have limited emotional intelligence and empathy, which can lead to output that is insensitive or inappropriate	2.24 (1.05)	2.27 (1.00)	t = 0.640	0.522
AI tools have limitations in their ability to handle complex tasks	2.06 (0.86)	2.24 (0.88)	t = 4.373	< 0.001**

t = independent t test.

** Significant at ≤ 0.001.

### Pattern of AI use in medical education and research among the studied population

**Fig. 2:**
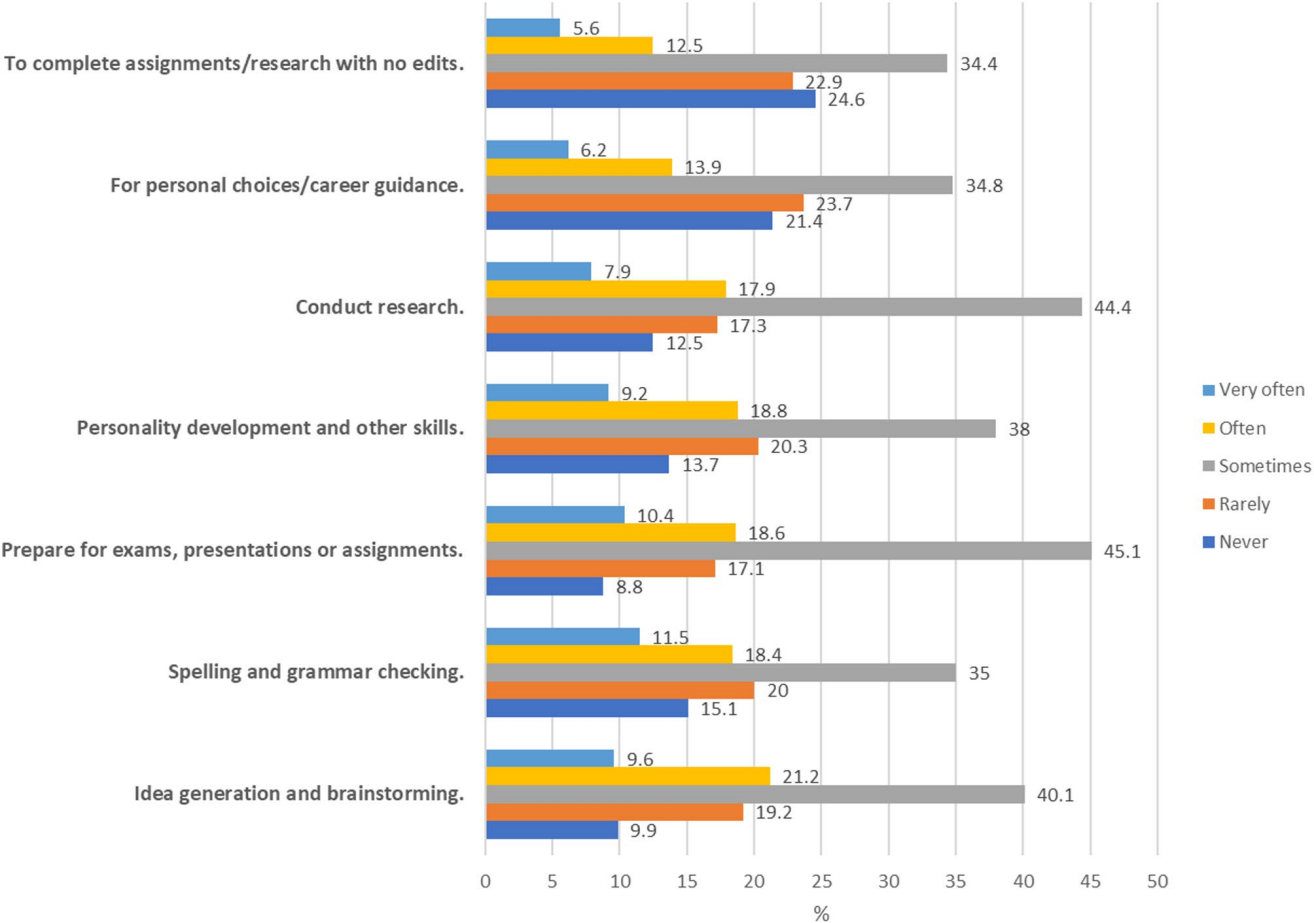
Bar chart showing the pattern of usage of Artificial Intelligence among medical students (*N* = 2765).

In the current study, medical students showed a statistically significant higher percentage of usage than staff in all items of the scale (*p* < 0.001). The most reported pattern of usage (rated as “very often” or “often”) of Artificial Intelligence among medical students was for idea generation and brainstorming (30.7%), followed by use for spelling and grammar checking (29.9%), then to prepare for exams, presentations, or assignments (29%). And the least reported pattern was using AI tools to complete assignments or research and submitting the output without modification (18.1%) (Table 5) (Fig. 2).

**Table 5:** Pattern of usage (combined “often” and “very often” responses) of Artificial Intelligence among the studied medical staff and students (*N* = 3265).

Pattern of usage of Artificial Intelligence		Medical staff (*N* = 500)	Medical students (*N* = 2765)	Test of significance	*p* value
I use Artificial Intelligence tools to complete assignments/research and then turn it in as is, with no edits		68 (13.6%)	501 (18.1%)	Z = 2.452	0.014*
I use Artificial Intelligence tools for personal choices/career guidance		80 (16.0%)	556 (20.1%)	Z = 2.135	0.033*
I use Artificial Intelligence tools to conduct research		82 (16.4%)	712 (25.8%)	Z = 4.485	< 0.001**
I use Artificial Intelligence technologies for personality development and other skills		74 (14.8%)	772 (27.9%)	Z = 6.162	< 0.001**
I use Artificial Intelligence tools to prepare for exams, presentations/ or assignments		77 (15.4%)	802 (29.0%)	Z = 6.312	< 0.001**
I use Artificial Intelligence for spelling and grammar checking		107 (21.4%)	828 (29.9%)	Z = 3.890	< 0.001**
I use Artificial Intelligence for idea generation and brainstorming		75 (15.0%)	849 (30.7%)	Z = 7.174	< 0.001**
**Total pattern of usage score**	**Mean (SD)**	**17.45 (5.74)**	**19.89 (5.22)**	**t = 8.875**	< 0.001**

*Significant at ≤ 0.05. **Highly significant at ≤ 0.001.

Z = Z for two proportions, t= independent t test.

### Concerns regarding AI use in medical education, research, and medical practice

On a 3-point scale (1 = Disagree, 3 = Agree), most mean scores ranged between 2.16 and 2.55, indicating moderate agreement with the listed concerns. For educational concerns, medical staff expressed statistically significant higher concerns than students, as reflected in their higher mean score in that using AI to complete assignments/research undermines the value of university education (*p* = 0.023) and that AI could introduce bias and fairness issues (*p* = 0.003). For medical practice concerns, medical staff expressed statistically significant higher agreement than students that AI devalues the medical profession (*p* = 0.003) and could affect equity in healthcare (*p* = 0.029). Most other items did not differ significantly between groups (Table 6).

**Table 6:** Concerns regarding the use of Artificial Intelligence in medical education and research among the study population.

	Medical staff (Mean (SD)	Medical students (Mean (SD)	Test of significance	*p* value
*Concerns regarding the use of Artificial Intelligence in medical education and research*				
Using AI technologies to complete assignments/research undermines the value of university education	2.35 (0.67)	2.28 (0.68)	t = 2.266	**0.023**
AI technologies will limit the opportunities to interact with others and socialize	2.46 (0.68)	2.43 (0.68)	t = 0.732	0.464
AI technologies will hinder the development of transferable skills such as teamwork, problem-solving, and leadership skills	2.43 (0.70)	2.37 (0.69)	t = 1.801	0.072
I have concerns related to privacy and scientific data security	2.48 (0.67)	2.49 (0.64)	t = 0.320	0.749
I have concerns about AI plagiarism and data fabrication	2.50 (0.67)	2.46 (0.65)	t = 1.286	0.198
I have concerns related to AI bias and fairness	2.48 (0.67)	2.38 (0.66)	t = 2.963	**0.003**
AI tools will have a negative impact on the scientific workforce	2.44 (0.65)	2.42 (0.67)	t = 0.712	0.477
I have Reproducibility and reliability concerns regarding AI-generated research	2.43 (0.66)	2.41 (0.63)	t = 0.887	0.375
I have concerns regarding the use of AI-powered cheating among undergraduate students	2.48 (0.65)	2.47 (0.66)	t = 0.273	0.785
*Concerns regarding the use of Artificial Intelligence in medical practice*				
The use of AI technologies will negatively affect the patient-physician relationship	2.47 (0.68)	2.40 (0.70)	t = 1.938	0.053
The use of AI technologies devalues the medical profession	2.26 (0.75)	2.16 (0.75)	t = 2.934	0.003**
The use of AI technologies reduces the humanistic aspect of the medical profession	2.41 (0.69)	2.35 (0.72)	t = 1.654	0.098
I have concerns related to privacy and patient data security: violations of professional confidentiality	2.51 (0.62)	2.46 (0.64)	t = 1.785	0.074
I have concerns related to AI bias and inaccuracy. If the data used to develop AI systems is incomplete or flawed, it could lead to inaccurate diagnoses or treatments	2.51 (0.64)	2.55 (0.60)	t = 1.263	0.207
I have concerns about technology failures. Like any technology, AI systems can fail or malfunction	2.51 (0.65)	2.54 (0.61)	t = 1.109	0.268
I have concerns about ethical and accountability issues. AI systems can’t make ethical judgments or consider the moral and social aspects that often play a role in healthcare decisions	2.53 (0.63)	2.51 (0.61)	t = 0.405	0.685
I have concerns about equity. While AI could increase access to healthcare, the costs associated with its use in personal medical devices could also do the opposite	2.54 (0.58)	2.47 (0.60)	t = 2.190	0.029*

*Significant at ≤ 0.05. **Highly significant at ≤ 0.001 t= independent t test.

Notably, both groups reported similarly high levels of worry regarding privacy violations, AI bias and inaccuracy, and the risk of technological failure, reflecting a broad recognition of the potential risks associated with AI deployment in healthcare systems. The lack of significant differences in most items suggests that concerns about the ethical, technical, and relational implications of AI are common across experience levels.

## Discussion

The influence of AI has drastically spread so that it is determined to become a pillar in the future medical world^16^. This national cross-sectional study represents the largest study conducted in Egypt to evaluate the knowledge, attitudes, and concerns regarding the use of AI among medical staff and students in Egypt and the second to compare medical students with faculty staff members, following the study conducted by Abokresha et al. (2025) at Sohag University^12^.

### Knowledge and attitude towards Artificial Intelligence in relation to sociodemographic characteristics of the studied population

The current study showed that a greater proportion of students (69.5%) than staff (57.6%) reported good perceived knowledge, with a highly statistically significant difference between both groups (*p* < 0.001). This agrees with Kansal et al.^17^. These findings may be attributed to greater exposure of younger generations to digital technologies and active engagement with online resources, while medical staff may have less time to explore new technologies outside their clinical and academic responsibilities, and some may face challenges in adapting to rapidly evolving digital tools. In the same context, male medical staff reported significantly higher perceived knowledge scores compared to females (5.22 vs. 4.83, *p* = 0.012). This was inconsistent with Serbaya et al., (2024), where there was no significant difference between male and female healthcare workers in Saudi Arabia regarding the mean knowledge score of AI^18^. This difference among staff may be explained by variations in access to or engagement with technology, professional roles, or confidence in self-reporting knowledge.

Regarding attitude, this study found almost equal mean attitude scores among both medical students and staff (35.13 ± 5.04 vs. 35.29 ± 5.29), which was against the findings of Kansal et al., (2022) where the percentage of doctors with a negative attitude towards AI was more than what was found in medical students^17^. Male participants had significantly higher attitude scores than females in both groups, which agreed with Heinrichs et al.^19^.

Both students and staff with good perceived knowledge demonstrated significantly higher attitude scores than those with poor knowledge. Despite these differences, the majority of both groups (80.4% of medical staff and 81.6% of medical students) expressed negative attitudes toward AI. The results of the present study were consistent with those of Khater et al.^2^. Perceived knowledge score and gender were significant predictors of attitude towards AI among medical staff while gender was a significant predictor of attitude scores among medical students which agreed with Allam et al. (2024) study and with Khan Rony et al.^11,20^.

### Perspectives on AI tools’ outputs among the study population

The most frequently reported concern in the current study was AI’s limited capacity to handle complex tasks (74% of staff and 66.1% of students), followed by concerns about limited emotional intelligence and empathy (69.4% and 63.8%) which agreed with Chan & Hu (2023). However, 90.8% of health professionals and 93.9% of health students in Al-Kahtani et al. (2025) study agreed that AI output reliability and accuracy are the most critical factors influencing AI adoption^15,21^.

Medical staff consistently reported higher agreement that AI can struggle with complex tasks, produce inaccurate or contextually inappropriate output, and display bias, reflecting their greater clinical exposure and first-hand experience with decision-making where accuracy, fairness, and contextual understanding are critical. Overall, the differences indicate that clinical experience strengthens recognition of specific AI risks, while students demonstrate broader but less experience-based concern. However, recent advances in software development and new generative AI solutions designed for fact-checking in the health and medical practice^22^, and to overcome the limits of static training data and the hallucination problem that is frequently seen in LLMs, TrumorGPT integrates graph-based retrieval-augmented generation (GraphRAG). GraphRAG entails using and accessing data from frequently updated semantic health knowledge graphs that include the most recent health and medical news, guaranteeing that TrumorGPT’s fact-checking is founded on the most recent data^23^.

Accordingly, structured education and training on AI applications, grounded in up-to-date medical evidence, are recommended for the studied medical staff and students.

### Pattern of use of AI tools

This study showed that about one-third of medical students use AI mainly for idea generation, proofreading, and academic preparation, with lower use for research and career guidance, and 18.1% reporting potentially problematic use, with a mean usage score of 19.89. Compared with other studies, AI use was highest for studying among undergraduates (64.4%), while Jordanian health profession students mainly used AI for grammar checking and research, with lower use for career advice and exam preparation, and a median practice score of 21/35^14,24^.

The total AI usage score in the following study was significantly higher among students than staff, these findings are consistent with Varshney et al.^25^. Students’ higher pattern of AI usage can be attributed to their heavier academic workload, stronger digital adaptability, and greater reliance on quick and accessible resources to support learning.

A noteworthy finding is the significantly higher percentage of students who admitted to using AI-generated work with no further editing, indicating a potential risk of overreliance that may compromise originality, academic integrity, and critical thinking development. The significantly higher total mean usage score among students further reinforces that AI has a stronger presence in the academic environment compared to clinical practice.

### Concerns of study participants regarding the integration of AI into educational and research contexts

The most prominent issues among medical staff were AI-related plagiarism and data fabrication (59.2%), followed by worries about privacy and scientific data security (58.6%), and concerns regarding AI bias and fairness (57.8%). In comparison with AlZahrani et al. (2025), the most frequently reported concerns were accuracy and reliability of AI technologies (21.2%), followed by worries about over-reliance on technology (20.5%) and issues related to data privacy and security (20.0%)^26^.

Medical students were primarily concerned about privacy and cheating, followed by plagiarism, reduced social interaction, career impact, skill development, reliability and bias issues, and the potential devaluation of university education. Meanwhile In Wobo et al.^27^, the most reported limitation of AI was ethical concerns, followed by lack of human interaction and overreliance on technology.

Mehrabi et al.^28^ highlighted important ethical issues associated with using AI in assessment processes. Fischer et al.^29^ noted that students may attempt to submit AI-generated answers as their own. Williams^30^ pointed out that many international students with diverse academic norms and ethical expectations, may not fully understand what constitutes academic misconduct.

In the present study, medical staff demonstrated slightly higher total concern score regarding the use of AI in medical education and research compared with students, with the difference approaching significance (*p* = 0.055) which was in line with Abokresha et al.^12^.

Although not statistically significant, this pattern indicates that educators may require more reassurance, guidance, and structured policy frameworks to confidently adopt AI-supported practices and highlights the need for institutional policies and training programs that not only regulate AI use but also balance innovation with academic integrity and skill development.

### Concerns of study participants regarding AI use in medical practice

Medical staff were significantly more likely to believe that AI devalues the medical profession and may undermine equity in healthcare access. In contrast, students—who are still forming their professional identity—expressed slightly lower concerns in these areas.

Several studies highlight significant concerns among healthcare providers regarding AI use. In Alsaedi et al.^31^, the main worry was job replacement, followed by fears that AI could justify staff reductions. Abdelwanis et al.^32^ identified data privacy as the most frequently reported issue and emphasized potential harm to patient–physician interaction. Similarly, Akudjedu et al.^33^ noted concerns about loss of the human aspect of care and weakened trust and rapport. Sangers et al.^34^ reported barriers such as accuracy concerns, limited integration of clinical judgment, lack of transparency, and risks of biased data leading to health disparities, alongside fears of role replacement. Quinn et al.^35^ warned about over-reliance on AI increasing vulnerability to technical failures and cyberattacks. Additionally, Mache et al.^36^ raised concerns about data quality, algorithmic bias, and accountability for AI-related errors.

The findings indicate that both medical staff and students share significant ethical and equity-related concerns regarding AI integration in healthcare. In the current study, 59.8% of medical staff expressed worry that AI systems cannot make ethical judgments or account for the moral and social dimensions of clinical decision-making, a concern supported by Farhud et al.^37^ who emphasized the potential loss of empathy and human compassion in AI-assisted care. Similarly, students have reported notable ethical apprehensions, as highlighted by Jackson et al.^7^, reflecting shared anxiety about the moral implications of AI use in medicine. Concerns about equity and affordability were also common across both groups. More than half of the medical staff (58.4%) were worried that AI-related costs—particularly in personal medical devices—could limit fair access to care, echoing findings from Eltorai et al.^38^ Likewise, students in Jordan, as reported by Al-Qerem et al.^14^ feared that expensive AI tools might widen disparities in healthcare and education. Furthermore, Khater et al.^2^ found that students recognized both the benefits and challenges of AI, demonstrating balanced but cautious attitudes.

This study results show a significant, evidence-based gap between the actual usage of AI by staff and students and the preparedness of medical education institutions to control that use. personnel are concentrating on long-term career and societal consequences while students are more concerned with completing their courses. The near-significant difference in concern scores (*p* = 0.055) should not be ignored, and students must be included in preparing policy change through joint student-staff committees to draft AI guidelines, otherwise it will be disregarded or evaded. These findings open several clear, high-priority research questions that must transcend prevalence of AI usage patterns and concerns to comprehend the cognitive and professional repercussions of overreliance and to evaluate interventions that preserve critical thinking while leveraging AI’s advantages in both medical education and practice among medical students and staff members.

### Strengths and limitations of the study

The study may have some limitations; first, the cross-sectional design precludes establishing causal relationships between knowledge, attitudes, and concerns. And the use of convenient and snowball sampling techniques may introduce selection bias and limit the generalizability.

Despite these limitations, the study demonstrates several notable strengths, such as timeliness and relevance: Artificial Intelligence is rapidly evolving, and this national snapshot captures current perceptions, attitudes, and ethical concerns during a transformative period, and provides comprehensive baseline data for future longitudinal studies. Furthermore, the large sample size and national nature of the study enhance representativeness, reflect broader diversity in academic settings, and strengthen generalizability within the country. And the involvement of both medical staff and students provides multi- level academic perspectives, rather than a single-group view.

## Conclusions

The study revealed that negative attitudes were prevalent among both medical staff and students. Satisfactory perceived knowledge was a strong predictor of positive attitudes. Students reported significantly higher AI usage in medical education; however, there were faulty patterns of use among them. Medical staff expressed stronger concerns related to the use of AI in medical education and both groups expressed concerns regarding the use of AI in medical practice; however, medical staff expressed significantly greater concerns that AI use devalues the medical profession, and equity in care provision.

The results of this study highlight the critical need for a comprehensive approach to medical education and practice through the following steps:

- Raising awareness of AI evidence-based benefits and risks, through seminars, real-world case studies, structured AI literacy programs, and continuing professional development activities.
- Integration of structured AI education into medical curricula.
- Addressing ethical and legal concerns and developing clear institutional guidelines and standards.
- Fostering interdisciplinary collaboration.
- Suggesting further research on AI related risks in the medical field and how it can be combated.

Medical staff and students can benefit from AI technologies by addressing these issues, ensuring that AI technologies can enhance rather than complicate medical education and clinical practice.

### Statistical analysis

The collected data were cleaned and coded then tabulated and analyzed using SPSS version 25 software (SpssInc, Chicago, ILL Company). Categorical data were presented as numbers and percentages, while quantitative data were expressed as mean ±standard deviation (SD). Chi-squared test (χ^2^) was used to analyze categorical variables. The Z test (Z) was used for 2 proportions. Continuous data were tested for normality using visual inspection of histograms and normal Q-Q plots. An independent t-test (t) was used to analyze normal variables among 2 independent groups, while ANOVA was used for more than 2 independent groups. Parametric correlations were assessed by Pearson’s correlation coefficient (r). Multiple linear regression analysis was performed to identify the independent predictors of knowledge and attitude scores. Assumption of linearity, normality, independence of residuals, and absence of multicollinearity (VIF < 5) were checked and met. A multiple linear regression analysis was done to look for predictors of knowledge and attitude score among medical staff and students. All independent variables were included in the regression analysis based on their theoretical relevance. The concern scores were included based on theoretical relevance to the outcome. The full model has been reported, including unstandardized B coefficient, SE, 95% CI, p-values, overall model fit (adjusted R²), and F-statistic to indicate explained variance. The accepted level of significance in this work was stated at 0.05 (*p* ≤ 0.05 was considered significant).

## Data Availability

All data that support the research finding are available from the corresponding author on request from the editor.
